# Oxidative stress biomarkers of neonates in Saudi population: An observational study

**DOI:** 10.1097/MD.0000000000041926

**Published:** 2025-03-21

**Authors:** Sobhy M. Yakout, Malak A. AlSubhi, Syed D. Hussain, Abdullah M. Alnaami, Abir A. Alamro, Nasser M. Al-Daghri

**Affiliations:** a Chair for Biomarkers of Chronic Diseases, Department of Biochemistry, College of Science, King Saud University, Riyadh, Saudi Arabia.

**Keywords:** antioxidant status, catalase, glutathione, glutathione peroxidase, oxidative stress, Saudi neonates, superoxide dismutase

## Abstract

Oxidative stress results from an imbalance between free radical production and antioxidant defense, significantly impacting neonatal health, particularly in preterm infants with immature antioxidant systems. This study aims to assess oxidative stress in Saudi neonates by measuring key antioxidants, both enzymatic (superoxide dismutase, catalase, glutathione peroxidase) and nonenzymatic (glutathione, bilirubin, uric acid), and comparing them across sex (male vs female) and term status (full term vs preterm). A total of 110 Saudi neonates (55 normal neonates and 55 preterm neonates; 52 females and 58 males) were included in this study. The gestational age of preterm neonates ranged from 28 to 36 weeks, with a mean of 32 weeks. Serum samples were retrieved from the chair for biomarkers of chronic diseases BioBank. Ethical approval was obtained from the College of Medicine, King Saud University. GSH levels were higher in preterm neonates compared to normal neonates (16.4 vs 11.0 µmol/L, *P* < .001), and uric acid levels were higher in normal neonates compared to preterm neonates (246.2 vs 206.2 µmol/L, *P* < .015). SOD1 levels were higher in preterm neonates compared to normal neonates (291.5 vs 225.4 ng/mL, *P* < .040). In terms of both term and sex of neonates, GSH levels were higher in preterm female neonates compared to normal female neonates (16.8 vs 13.8 µmol/L, *P* < .054), and in preterm male neonates compared to normal male neonates (16.4 vs 9.2 µmol/L, *P* < .001). SOD1 levels were higher in preterm male neonates compared to normal male neonates (300.1 vs 198.8 ng/mL, *P* < .038), and uric acid levels were higher in normal male neonates compared to preterm male neonates (243.9 vs 200.1 µmol/L, *P* < .011). GPx-1 levels were higher in preterm neonates compared to normal neonates (14.6 vs 7.9 ng/mL, *P* < .006). There are no differences in antioxidant parameters between female and male neonates. However, some antioxidants differ between preterm and normal neonates. The comparison according to both sex and term status also showed differences in some antioxidant parameters.

## 1. Introduction

Oxidative stress (OS) is an imbalance between free radical production and antioxidant defense, leading to excessive free radicals that disrupt redox signaling and physiological functions. This involves reactive oxygen species (ROS) and reactive nitrogen species, contributing to various diseases. OS can be generated both internally and externally, influenced by factors like diet, environmental chemicals, smoking, and aging. Measurement of OS in diseases involves analyzing DNA, protein, and lipid oxidation, with antioxidants such as glutathione protecting cells from free radical damage.^[1–6]^

The “free-radical theory,” proposed by Harmans in 1956, suggests that ROS produced during aerobic oxidation can harm cells, leading to cell death. Mitochondria are a major source of these radicals, with components of the electron transport chain and enzymes like α-ketoglutarate dehydrogenase contributing to ROS formation. External factors like ionizing radiation and cigarette smoke also contribute to ROS generation.^[7–11]^

ROS are involved in physiological processes and pathological processes of diseases like Alzheimer, Parkinson, diabetes mellitus, cardiovascular diseases, and cancer. In diabetes, OS contributes to vascular complications and insulin resistance, while in cancer, increased ROS levels link to chronic inflammation and DNA mutations.^[12–16]^

OS during pregnancy can affect fetal development, birth outcomes, and lead to diseases later in life. The placenta plays a central role in managing stress during pregnancy, with complications like preeclampsia and fetal growth restriction being potential outcomes. OS also impacts fertility, affecting sperm motility and oocyte quality.^[17–23]^

In preterm infants, conditions like bronchopulmonary dysplasia (BPD), necrotizing enterocolitis (NEC), and retinopathy of prematurity (ROP) are influenced by OS. BPD involves impaired lung function, NEC is characterized by inflammation in the gastrointestinal tract, and ROP involves abnormal retinal blood vessel growth.^[24–28]^

Antioxidant biomarkers, including enzymatic and nonenzymatic antioxidants like glutathione, total bilirubin, uric acid, ascorbic acid, and vitamin E, are crucial for assessing OS status. Glutathione is the most abundant intracellular antioxidant, while total bilirubin and uric acid act as potent antioxidants in plasma. Ascorbic acid and vitamin E also **significantly protect** against oxidative damage. Enzymatic antioxidants such as superoxide dismutase, catalase, and glutathione peroxidase are important for detoxifying superoxide anion and hydrogen peroxide.^[29–33]^ These antioxidants are essential for a comprehensive assessment of OS status.

Studies across various regions, including North America^[31,34,35]^ have extensively explored OS in neonates, focusing on its impact on different neonatal conditions and the role of various biomarkers. However, research specifically in Saudi Arabia is limited. Our study aims to fill this gap by examining the OS status in Saudi neonates, differentiated by sex (male vs female) and term status (full term vs preterm), through the measurement of both enzymatic (superoxide dismutase, catalase, glutathione peroxidase) and non-enzymatic (glutathione, bilirubin, uric acid) antioxidant biomarkers. This will provide crucial insights specific to the Saudi neonatal population.

## 2. Methodology

### 2.1. Subjects selection

**Figure 1.**
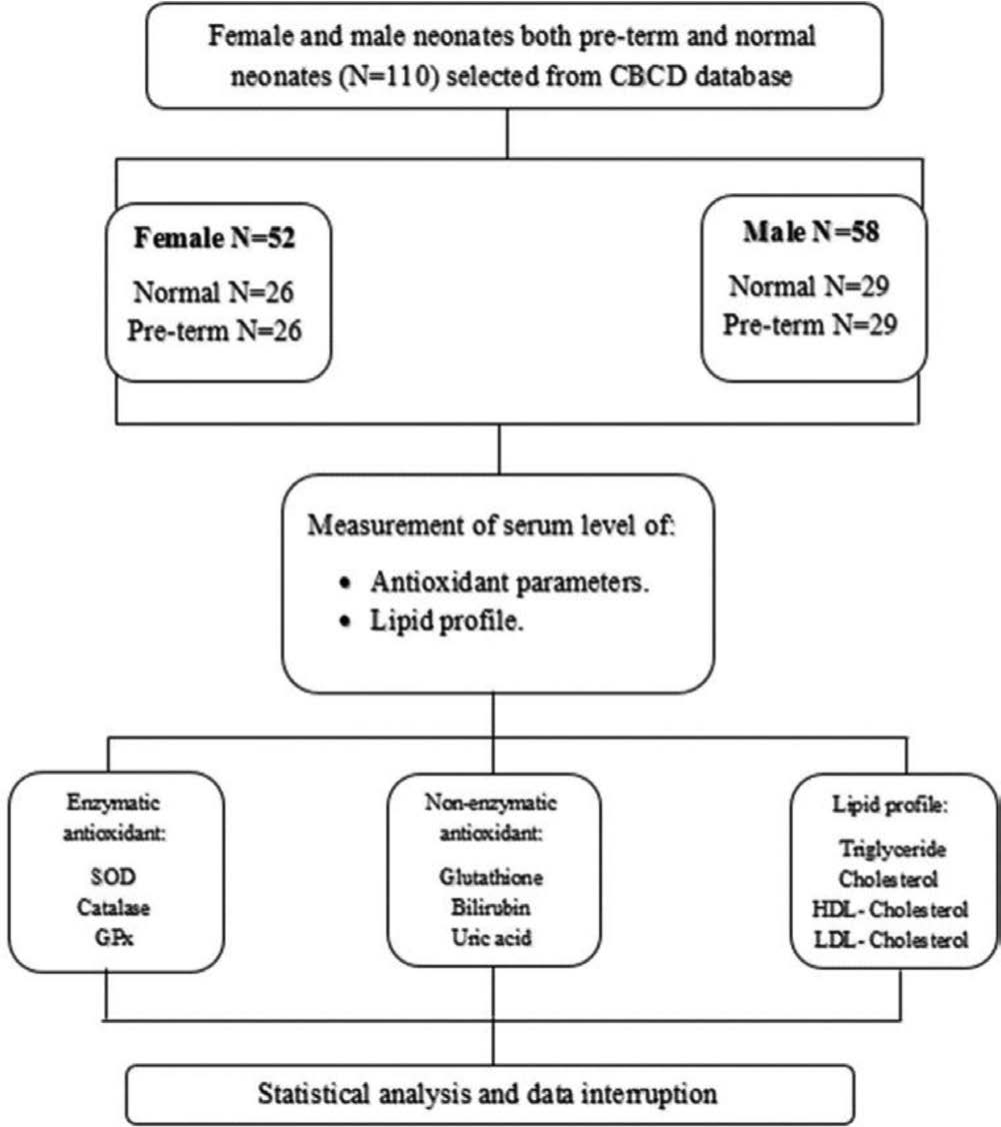
Schematic diagram of the methodology.

A total of 110 Saudi neonates both male and female were included in this study. Subjects were divided into 2 groups depending on the term status: the first group was preterm (n = 55) and term (n = 55). Figure 1 shows the schematic diagram of the methods. The gestational age in weeks for the preterm neonates was recorded. The gestational age of the preterm neonates ranged from 28 to 36 weeks, with a mean gestational age of 32 weeks. The serum sample of subjects was retrieved from the Chair for Biomarkers of Chronic Diseases BioBank, Biochemistry department, College of Science, King Saud University, Riyadh, Saudi Arabia along with the clinical history of the parent including the causes of preterm delivery. The causes of preterm delivery in our study cohort included maternal factors such as high blood pressure, gestational diabetes mellitus, infections, and other complications. This study’s ethical approval was obtained from the Institutional Review Board (IRB) of the College of Medicine, King Saud University, Riyadh, Saudi Arabia (IRB Approval No. E-21-5660). Written informed consent was obtained from the parents or guardians of all neonates included in the study.

### 2.2. Sample analysis

#### 2.2.1. Determination of glucose and lipid profile

Glucose, total cholesterol, High density lipoprotein cholesterol (HDL-cholesterol), and, triglyceride (TAG) levels in neonates’ serum samples were measured using a Konelab auto analyzer. The Konelab instrument measures the serum concentration of many parameters by the enzymatic colorimetric reaction in which the product formed during the reaction will absorb the light at a specific wavelength thus reflecting the concentration of the desired parameter. This was done with the help of a kit specific to each test obtained from ThermoFisher, Vantaa, Finland.

#### 2.2.2. Nonenzymatic antioxidants parameters measurements

The serum glutathione (GSH), total bilirubin, and uric acid are measured colorimetrically on a Konelab instrument using commercial kits For GSH and its oxidized form GSSG, the glutathione colorimetric detection kit (Invitrogen, Waltham) is used, where the reaction of GSH with a substrate forms a colored product, measured at 405 nm to determine its concentration. Total bilirubin is quantified using the Bilirubin total (NBD) kit (ThermoFisher), where bilirubin forms azobilirubin through a reaction with provided reagents. The color intensity of azobilirubin, measured at 540 nm, indicates the bilirubin levels in the sample. Uric Acid measurement employs the Uric Acid (AOX) kit (ThermoFisher), where uric acid is transformed into allantoin and hydrogen peroxide, leading to a blue-violet dye formation. The dye’s intensity, measured at 540 nm, corresponds to the uric acid concentration in the sample.

#### 2.2.3. Enzymatic antioxidants parameters measurements

The concentrations of superoxide dismutase 1 (SOD1), catalase, and glutathione peroxidase-1 (GPx-1) in serum samples were determined using specialized kits. SOD1 was measured with the Human Cu/Zn SOD ELISA Kit, involving a reaction in a 96-well plate that led to a measurable color change at 450 nm. Catalase activity was assessed with the Catalase Colorimetric Activity Kit (Invitrogen), where a color change in the presence of hydrogen peroxide indicated catalase levels, measured at 560 nm. GPx-1 levels were quantified using a Sandwich ELISA Kit (Antibodies online, Aachen, Germany), with a colorimetric reaction in a 96-well plate measured at 450 nm after adding specific reagents.

### 2.3. Statistical analysis

SPSS version 23.0, IBM (Armonk) was used to analyze all data. The continuous data for normal variables is presented as (mean ± standard deviation) while the non-normal variables are presented as (median [1st Quartile–3rd Quartile]). *P*-values are obtained from the independent sample *t*-test and Mann–Whitney *U*-test for normal and non-normal variables respectively. For the categorical variables data presented as frequencies (%), the Chi-square test was used to obtain *P*-values for categorical variables. The correlation between study parameters in all subjects is presented as the Spearman correlation coefficient (*R*). *P*-value < .05 is considered significant. Median (25th percentile—75% percentile).

## 3. Results

In the study of 110 Saudi neonates, comprising 55 normal and 55 preterm, Table 1 detailed descriptive statistics, showing no significant differences in maternal parameters between the groups. However, in neonatal parameters, GSH levels were significantly higher in preterm neonates (16.4 µmol/L) compared to normal neonates (11.0 µmol/L, *P* < .001). Uric acid concentrations were higher in normal neonates (246.2 µmol/L) than in preterm neonates (206.2 µmol/L, *P* < .015). Additionally, SOD1 levels were significantly elevated in preterm neonates (291.5 ng/mL) compared to normal ones (225.4 ng/mL, *P* < .040).

**Table 1:** Descriptive statistics according to term status.

Parameters	Normal	Preterm	*P*-values
N	55	55	
*Maternal parameters*			
Maternal age (yr)	28.8 ± 3.6	30.8 ± 6.5	.081
Maternal weight (kg)	77.3 ± 17.1	79.7 ± 13.3	.469
Parity #	2.0 (0.0–3.0)	2.0 (1.0–3.0)	.704
Exercise	4 (7.3)	3 (5.5)	1.000
High blood pressure	1 (1.8)	2 (3.6)	1.000
High cholesterol	2 (3.6)	1 (1.8)	1.000
Gestational diabetes mellitus	2 (3.6)	6 (10.9)	.271
*Neonatal parameters*			
Birthweight (kg)	3.1 ± 0.4	3.1 ± 0.5	.573
Gender (male/female)	29/26	29/26	1.000
Normal delivery	55 (100.0)	54 (98.2)	1.000
GSH (µmol/L)#	11.0 (7.2–15.1)	16.4 (13.0–17.8)	<.001
Cholesterol (mmol/L)	1.8 ± 0.5	1.7 ± 0.5	.174
Glucose (mmol/L)	6.7 ± 1.6	6.2 ± 1.5	.116
HDL-cholesterol (mmol/L)	0.4 ± 0.1	0.4 ± 0.1	.680
LDL-cholesterol (mmol/L)	0.8 ± 0.4	0.7 ± 0.4	.229
Triglycerides (mmol/L)	1.3 ± 0.5	1.3 ± 0.3	.441
Total bilirubin (µmol/L) #	31.1 (26.4–37.8)	30.7 (26.2–37.3)	.480
Uric acid (µmol/L) #	246.2 (204.5–274.6)	206.2 (175.8–247.7)	.015
SOD1 (ng/mL)	225.4 (162.6–347.0)	291.5 (217.2–417.2)	.040
Catalase2 (U/mL)	22.3 ± 3.1	22.7 ± 2.0	.450
GPx-1 (ng/mL)	11.0 (4.4–17.1)	12.0 (9.0–19.4)	.192

Note: Data presented as Mean ± SD for normal and Median (1st Quartile–3rd Quartile) for non-normal variables. # indicates non-normal variables. *P*-values are obtained from independent sample *t*-test and Mann–Whitney *U*-test for normal and non-normal variables respectively. For categorical variables data presented as N (%). Chi-square test was used to obtain *P*-values for categorical variables. *P*-value < .05 considered significant. Median (25th percentile–75% percentile).

Abbreviations: GPx-1 = glutathione peroxidase-1, GSH = glutathione, HDL-cholesterol = high density lipoprotein cholesterol, LDL-cholesterol = low-density lipoprotein cholesterol, SOD 1 = superoxide dismutase 1.

Analyzing neonatal parameters based on term and gender (Table 2), there is a significant difference was found in specific neonatal biomarkers, while maternal parameters showed no significant differences. For GSH concentrations, preterm female neonates had higher levels (16.8 µmol/L) compared to normal neonates (13.8 µmol/L, *P* < .054), a pattern also observed in males with preterm neonates at 16.4 µmol/L versus 9.2 µmol/L in normal neonates (*P* < .001). SOD1 levels were significantly higher in preterm male neonates (300.1 ng/mL) compared to normal male neonates (198.8 ng/mL, *P* < .038). In contrast, uric acid concentrations were higher in normal male neonates (243.9 µmol/L) than in preterm ones (200.1 µmol/L, *P* < .011). Among female neonates, GPx-1 levels were notably higher in preterm neonates (14.6 ng/mL) compared to normal neonates (7.9 ng/mL, *P* < .006).

**Table 2:** Descriptive statistics according to gender and term status.

	Female (N = 52)			Male (N = 58)		
	Normal (N = 26)	Preterm (N = 26)	*P*-values	Normal (N = 29)	Preterm (N = 29)	*P*-values
*Maternal parameters*						
Age	29.0 ± 3.6	31.6 ± 6.0	.104	28.6 ± 3.7	30.1 ± 6.9	.405
Weight	80.0 ± 16.7	80.2 ± 14.8	.967	74.6 ± 17.4	79.2 ± 12.2	.298
Parity #	2.0 (1.0–3.0)	2.0 (0.0–5.0)	.787	2.0 (2.0–3.0)	2.0 (1.0–2.7)	.094
Exercise	3 (11.5)	1 (3.8)	.610	1 (3.4)	2 (6.9)	1.000
High BP	1 (3.8)	0	1.000	0	2 (6.9)	.491
High cholesterol	2 (7.7)	0	.490	0	1 (3.4)	1.000
Gestational diabetes mellitus	1 (3.8)	3 (11.5)	.610	1 (3.4)	3 (10.3)	.611
*Neonatal parameters*						
Mode of delivery (normal)	26 (100.0)	26 (100.0)	–	29 (100.0)	28 (96.6)	1.000
Birthweight	3.1 ± 0.4	3.1 ± 0.4	.887	3.2 ± 0.4	3.2 ± 0.4	.923
GSH (µmol/L) #	13.8 (10.5–15.1)	16.8 (9.0–17.7)	.054	9.2 (6.4–13.6)	16.4 (13.4–17.9)	<.001
Total cholesterol (mmol/L)	1.9 ± 0.6	1.7 ± 0.6	.417	1.8 ± 0.4	1.7 ± 0.5	.258
Glucose (mmol/L)	7.0 ± 1.8	6.2 ± 1.4	.114	6.5 ± 1.3	6.2 ± 1.5	.567
HDL-cholesterol (mmol/L)	0.4 ± 0.1	0.4 ± 0.1	.816	0.4 ± 0.1	0.4 ± 0.1	.159
LDL-cholesterol (mmol/L)	0.8 ± 0.5	0.7 ± 0.5	.586	0.8 ± 0.4	0.7 ± 0.4	.234
Triglycerides (mmol/L)	1.4 ± 0.4	1.2 ± 0.3	.063	1.3 ± 0.5	1.3 ± 0.3	.571
Total bilirubin (µmol/L)	31.2 (26.2–37.7)	29.0 (26.6–37.3)	.660	31.1 (26.5–37.8)	31.3 (26.0–36.2)	.715
Uric acid (µmol/L)	247.8 (214.6–273.9)	220.6 (180.3–267.7)	.153	243.9 (203.5–274.5)	200.1 (159.5–221.4)	.011
SOD1 (ng/mL)	277.8 (185.2–438.0)	291.5 (224.8–481.2)	.329	198.8 (114.9–315.2)	300.1 (205.9–396.8)	.038
Catalase2 (U/mL)	22.0 ± 2.9	23.0 ± 2.2	.175	22.6 ± 3.4	22.4 ± 1.8	.777
GPx-1 (ng/mL)	7.9 (3.4–17.1)	14.6 (11.9–21.1)	.006	11.6 (6.7–17.1)	9.4 (7.5–12.1)	.230

Note: Data presented as Mean ± SD for normal and Median (1st Quartile–3rd Quartile) for non-normal variables. # indicates non-normal variables. *P*-values are obtained from independent sample *t*-test and Mann–Whitney *U*-test for normal and non-normal variables respectively. For categorical variables data presented as N(%). Chi-square test was used to obtain *P*-values for categorical variables. *P*-value < .05 considered significant. Median (25th percentile–75% percentile).

Abbreviations: GPx-1 = glutathione peroxidase-1, GSH = glutathione, HDL-cholesterol = high density lipoprotein cholesterol, LDL-cholesterol = low-density lipoprotein cholesterol, SOD1 = superoxide dismutase 1.

**Figure 2.**
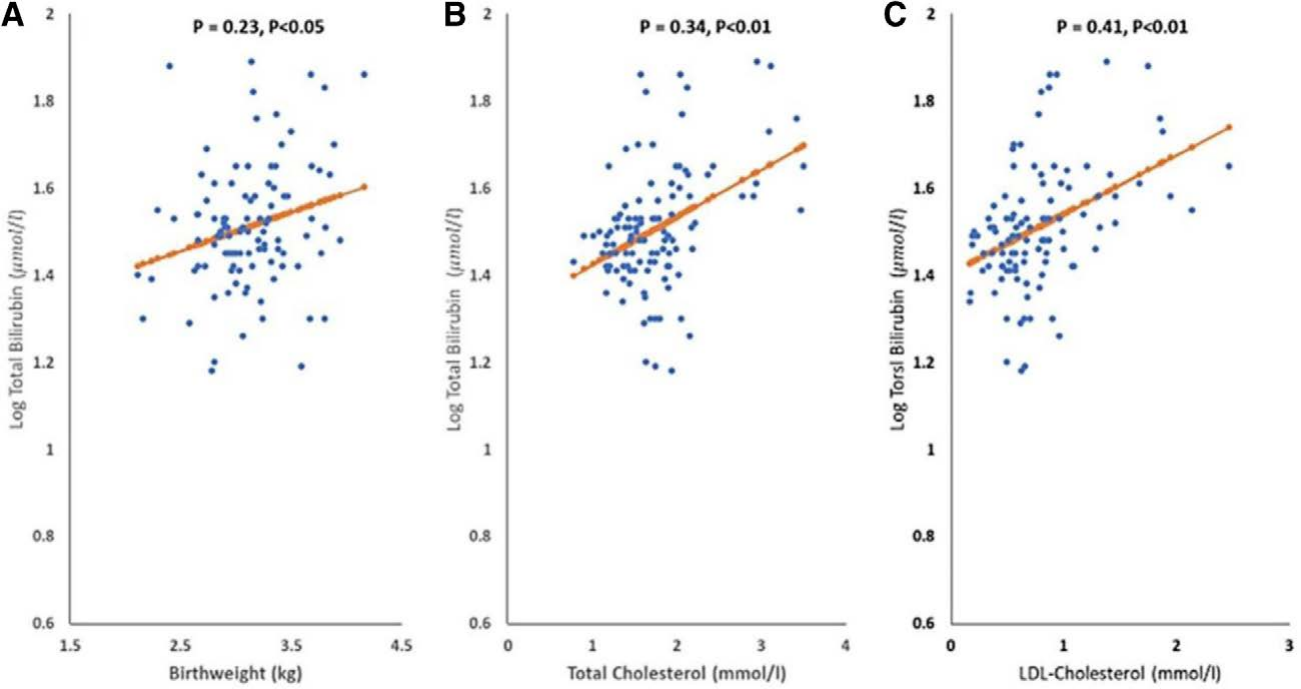
Correlation between (A) bilirubin (µmol/L) and birthweight (kg) at baseline in all subjects (B) bilirubin (µmol/L) concentration and total cholesterol (mmol/L) at baseline in all subjects (C) bilirubin (µmol/L) and LDL-cholesterol (mmol/L) at baseline in all subjects.

**Figure 3.**
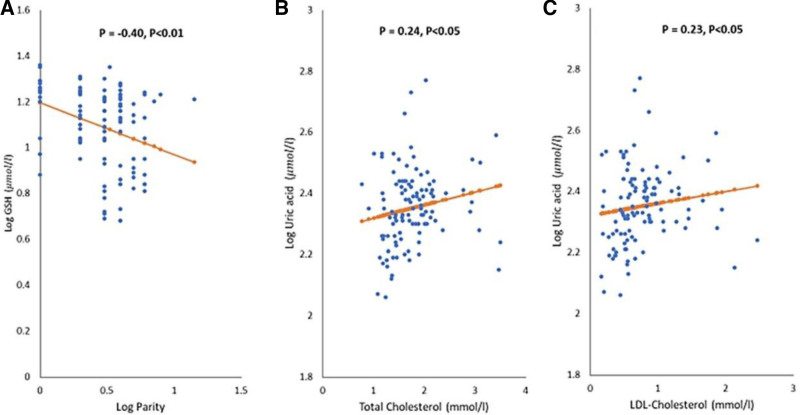
Correlation (A) between GSH (µmol/L) concentration and Parity at baseline in all subjects (B) between uric acid (µmol/L) and total Cholesterol (mmol/L) at baseline in all subjects (C) between uric acid (µmol/L) and LDL-Cholesterol (mmol/L) at baseline in all subjects.

In a study involving 110 neonates, half normal and half preterm, correlations were analyzed for various parameters. Total Bilirubin demonstrated direct correlations with birthweight, cholesterol, and Low-density lipoprotein cholesterol (LDL-Cholesterol), as shown in Figure 2, with slight variations between normal and preterm groups. GSH was inversely correlated with Parity (Fig. 3A). Uric acid had direct correlations with cholesterol and LDL-Cholesterol (Fig. 3B and C) and an inverse correlation with SOD1, with distinct differences between preterm and normal neonates. SOD1’s correlation patterns also differed between the 2 groups.

In a gender-specific study of neonates, GSH showed varying correlations: inversely with parity and catalase, and positively with birthweight, more so in preterm than in normal infants. Total Bilirubin consistently correlated with cholesterol markers, while Uric acid and SOD1 presented mixed correlation patterns that diverged between preterm and normal neonates, including direct associations with HDL-Cholesterol in preterm females and inverse ones with GSH in normal females. Conversely, in males, SOD1’s correlations were specific to neonate term status, with Catalase inversely related to GSH across the board but showing additional correlations only in preterm males. GPx-1 exhibited correlations with HDL-Cholesterol in normal females but was non-significant in all male subjects.

## 4. Discussions

In our study, we aimed to delineate the OS status in Saudi neonates, stratified by sex and term status, through the evaluation of both enzymatic and non-enzymatic antioxidants in umbilical cord blood samples. This investigation stands as a novel approach in its comprehensive inclusion of multiple antioxidant parameters, facilitating a more nuanced understanding of OS mechanisms in neonates.

Our findings indicate an elevation of GSH and SOD1 levels in preterm neonates, with term neonates showing increased uric acid levels. Notably, no discernible differences were observed between male and female neonates. The elevated GSH levels in preterm neonates, consistent with the observations of Moore et al (2018), could be indicative of a dysregulated antioxidant system, potentially due to the specific ROS prevalent in this demographic.^[31]^ The results suggest that the timing of sample collection and the methodologies employed in GSH quantification may significantly impact outcomes, as supported by Rook et al (2010) and Giuffrè et al (2015).^[23,36]^

Conversely, the increased uric acid levels noted in term neonates echo the findings of Moore et al (2018) and stand in contrast to Basu et al (2009), who reported elevated levels in preterm neonates’ urine samples.^[31,37]^ This discrepancy underscores the influence of methodological variables, including sample type and assay technique, and highlights the need for standardization in future research.

The study also revealed a previously unreported increase in SOD1 concentration among preterm neonates. Given the lack of analogous findings in existing literature, this could suggest a unique genetic predisposition within the Saudi population that merits further genetic and molecular investigation.^[38–40]^

The correlation analyses presented in this study provide insights into the complex interplay between various antioxidants and OS. The inverse relationship observed between GSH and catalase across all subjects implies an overarching dysregulation of the antioxidant system in neonates, which aligns with the findings of Solevåg et al (2019) and Agostoni et al (1980).^[41,42]^ The gender-specific direct correlation between GSH and SOD1 in male subjects, in particular, warrants further investigation to elucidate the underlying biological mechanisms, as it may reflect sex-dependent regulatory processes.^[43]^

Despite the robust findings, the study is not without limitations. The absence of precise gestational age data for preterm neonates poses a constraint on the granularity of our analysis. Therefore, future studies are encouraged to record detailed perinatal data to enhance the precision of OS assessments. Moreover, we advocate for additional research to explore the parental molecular history’s impact on neonatal outcomes, which could pave the way for innovative antioxidant-based therapeutic strategies in neonatal care.

## 5. Conclusion

In this study, we evaluate the OS status of Saudi neonates according to their sex and term status by measuring enzymatic and non-enzymatic antioxidants. The result shows there are no differences in all parameters between female and male. The result according to status shows that preterm neonates have GSH concentrations and SOD1 levels higher than normal neonates. While, uric acid concentration is higher in normal neonates compared to preterm neonates. According to both status and sex, In preterm female neonates, GPx-1 levels were higher than in normal female neonates, in preterm male neonates SOD1 levels higher than in male normal neonates, in normal male neonates uric acid was higher than preterm male neonates and in both male and female preterm GSH concentrations higher than those normal neonates. We found a numerous correlation between study parameters. First of all, GSH inversely correlates with catalase in all neonates, uric acid inversely correlates with SOD1 in preterm neonates, GSH inversely correlates with SOD1 in female neonates, GSH directly correlates with SOD1 in male neonates finally, catalase inversely correlates with SOD1 in preterm male neonates.

## Acknowledgments

The authors are grateful to the Deanship of Scientific Research, King Saud University for funding this research project through the Vice Deanship of Scientific Research Chairs.

## Author contributions

**Conceptualization:** Nasser M. Al-Daghri.

**Formal analysis:** Syed D. Hussain.

**Funding acquisition:** Nasser M. Al-Daghri.

**Investigation:** Malak A. AlSubhi, Abdullah M. Alnaami, Abir A. Alamro.

**Methodology:** Malak A. AlSubhi, Abdullah M. Alnaami, Abir A. Alamro.

**Supervision:** Sobhy M. Yakout, Abir A. Alamro, Nasser M. Al-Daghri.

**Writing – original draft:** Sobhy M. Yakout.

**Writing – review & editing:** Malak A. AlSubhi, Syed D. Hussain, Abdullah M. Alnaami, Abir A. Alamro.
